# Can diffusion-weighted imaging predict tumor grade and expression of Ki-67 in breast cancer? A multicenter analysis

**DOI:** 10.1186/s13058-018-0991-1

**Published:** 2018-06-19

**Authors:** Alexey Surov, Paola Clauser, Yun-Woo Chang, Lihua Li, Laura Martincich, Savannah C. Partridge, Jin You Kim, Hans Jonas Meyer, Andreas Wienke

**Affiliations:** 1 Department of Diagnostic and Interventional Radiology, University of Leipzig, Liebigstrasse 20, 04103 Leipzig, Germany; 2 Department of Biomedical Imaging and Image-guided Therapy, Medical University of Vienna, Währinger Gürtel, 18-20 1090 Vienna, Austria; 3 Department of Radiology, Soonchunhyang University Hospital, 59 Daesakwan-ro, Yongsan-gu, Seoul, 140-743 Republic of Korea; 4 Institute of Biomedical Engineering and Instrumentation, Hangzhou Dianzi University, Hangzhou, China; 5 Unit of Radiology, Institute for Cancer Research and Treatment of Candiolo (IRCC), Strada Provinciale 142, 10060 Candiolo, Turin, Italy; 6 Department of Radiology, University of Washington, 825 Eastlake Avenue E, G2-600, Seattle, WA 98109 USA; 7 Department of Radiology, Pusan National University Hospital, Pusan National University School of Medicine and Medical Research Institute, 1-10, Ami-Dong, Seo-gu, Busan, 602-739 Korea; 8 Institute of Medical Epidemiology, Biostatistics, and Informatics, Martin-Luther-University Halle-Wittenberg, Magdeburger Strasse, 06097 Halle, Germany

**Keywords:** Breast cancer, ADC, DWI, Ki-67

## Abstract

**Background:**

Numerous studies have analyzed associations between apparent diffusion coefficient (ADC) and histopathological features such as Ki-67 proliferation index in breast cancer (BC), with mixed results. The purpose of this study was to perform a multicenter analysis to determine relationships between ADC and expression of Ki-67 and tumor grade in BC.

**Methods:**

For this study, data from six centers were acquired. The sample comprises 870 patients (all female; mean age, 52.6 ± 10.8 years). In every case, breast magnetic resonance imaging with diffusion-weighted imaging was performed. The comparison of ADC values in groups was performed by Mann-Whitney *U* test where the *p* values are adjusted for multiple testing (Bonferroni correction). The association between ADC and Ki-67 values was calculated by Spearman’s rank correlation coefficient. Sensitivity, specificity, negative and positive predictive values, accuracy, and AUC were calculated for the diagnostic procedures. ADC thresholds were chosen to maximize the Youden index.

**Results:**

Overall, data of 870 patients were acquired for this study. The mean ADC value of the tumors was 0.98 ± 0.22 × 10^− 3^ mm^2^ s^− 1^. ROC analysis showed that it is impossible to differentiate high/moderate grade tumors from grade 1 lesions using ADC values. Youden index identified a threshold ADC value of 1.03 with a sensitivity of 56.2% and specificity of 67.9%. The positive predictive value was 18.2%, and the negative predictive value was 92.4%. The level of the Ki-67 proliferation index was available for 845 patients. The mean value was 12.33 ± 21.77%. ADC correlated with weak statistical significant with expression of Ki-67 (*p* = − 0.202, *p* < 0.001). ROC analysis was performed to distinguish tumors with high proliferative potential from tumors with low expression of Ki-67 using ADC values. Youden index identified a threshold ADC value of 0.91 (sensitivity 64%, specificity 50%, positive predictive value 67.7%, negative predictive value 45.0%).

**Conclusions:**

ADC cannot be used as a surrogate marker for proliferation activity and/or for tumor grade in breast cancer.

## Background

Breast cancer (BC) is the most common noncutaneous malignancy among women, representing four in ten female cancer survivors in the United States [1]. Different imaging modalities such as mammography, ultrasound, and magnetic resonance imaging (MRI) play an essential role in the diagnosis and local staging of BC. According to the literature, imaging not only can document breast lesions but also can predict histopathological features of BC [2–5]. For instance, Seo et al. reported that the human epidermal growth factor receptor 2 (HER2)-positive subtype of BC was associated with a higher BI-RADS (Breast Imaging Reporting and Data System) category [2]. Some authors also indicated that several imaging features can provide information about proliferation potential or expression of Ki-67 in BC [5]. Szabo et al. reported that rim enhancement on dynamic MRI was associated with high expression of Ki-67 and poor prognosis of BC [5]. Furthermore, numerous studies analyzed associations between diffusion-weighted imaging (DWI) and histopathological features in BC, including associations between apparent diffusion coefficient (ADC) and expression of Ki-67 [4, 6, 7]. However, the reported data were mixed. Whereas some authors found significant correlations between ADC and Ki-67 in BC, other did not [8–13]. For example, Li et al. observed a moderate statistically significant correlation between ADC and Ki-67 (*r* = − 0.566, *p* = 0.025) [8]. Similar results were also reported by Mori et al. [9]. These authors suggested that ADC would be practical to use for estimating the Ki-67 labeling index [9]. However, Aydin et al. could find only a weak negative correlation between ADC and Ki-67-values in BC (*r* = − 0.279; *p* = 0.029) [10]. Finally, some authors did not identify statistically significant correlations between these parameters [11–13]. Similarly, data about relationships between tumor grade and ADC were also inconsistent. These facts question the possibility to use ADC as a surrogate marker for proliferation activity in BC in clinical practice. The purpose of the present study was to provide evidence-based data regarding associations between ADC and expression of Ki-67 as well tumor grade in BC.

## Methods

### Data acquisition and patients

For this study, the MEDLINE library was screened for associations between ADC and Ki-67 in BC up to December 2017. The following search words were used: “DWI or diffusion weighted imaging or diffusion-weighted imaging or ADC or apparent diffusion coefficient AND Ki-67 OR KI67 OR ki67 OR ki-67 OR mitotic index OR proliferation index OR MIB 1 OR MIB-1 OR mitosis index AND breast cancer OR breast carcinoma.” Overall, 41 items were identified.

In the next step, corresponding authors of all identified reports were contacted via email with a request to provide the data of the investigated patients, including the following for every case: age, precise histopathological diagnosis, tumor grade, mean ADC values, and Ki-67 index. Overall, six of them provided their data [14–19]. Data were provided from the following centers:

- Department of Medical and Biological Sciences, Institute of Diagnostic Radiology, Azienda Ospedaliero Universitaria Santa Maria della Misericordia, University of Udine, Udine, Italy (center 1)
- Department of Radiology, Soon Chun Hyang University Hospital, Seoul, Republic of Korea (center 2)
- Institute of Biomedical Engineering and Instrumentation, Hangzhou Dianzi University, Hangzhou, China (center 3)
- Unit of Radiology, Cancer Institute, Institute for Cancer Research and Treatment of Candiolo (IRCC), Turin, Italy (center 4)
- Department of Radiology, University of Washington, Seattle, WA, USA (center 5)
- Department of Radiology, Pusan National University Hospital, Pusan National University School of Medicine and Medical Research Institute, Busan, Republic of Korea (center 6)

The acquired sample comprises 870 patients (all female; mean age, 52.6 ± 10.8 years; median age, 52 years; range, 24–85 years). The patients had a variety of breast tumor histologic types (Table 1). In every case, breast MRI with DWI was performed with a clinical scanner (1.5 and 3.0 T) with dedicated breast radiofrequency coils. MRI equipment and imaging protocols varied across centers (Table 2).

**Table 1:** Analyzed tumors

Diagnosis	No.	%
DCIS	45	5.17
IDC	713	81.95
ILC	57	6.55
Combined IDC/ILC	9	1.03
Mucinous carcinoma	7	0.81
Tubular carcinoma	3	0.35
Metaplastic carcinoma	2	0.23
Unspecified	34	3.91
Total	870	100

*Abbreviations: DCIS* Ductal carcinoma in situ, *IDC* Invasive ductal carcinoma. *ILC* Invasive lobular carcinoma

**Table 2:** Patients and magnetic resonance imaging techniques

Centers	No. of patients	MRI equipment and field strength	DWI sequences and b-values
1	115	1.5-T scanner (MAGNETOM Avanto; Siemens Medical Systems, Erlangen, Germany)	Single-shot echo-planar sequence (TR/TE: 7100/84 ms); b-values: 0–1000 s/mm^2^
2	335	1.5-T scanner (Sonata; Siemens Medical Systems, Erlangen, Germany)	Single-shot echo-planar sequence (TR/TE: 5000/110 ms); b-values: 0–1000 s/mm^2^
3	82	3.0-T scanner (MAGNETOM Verio, Siemens Medical Systems, Erlangen, Germany)	Single-shot echo-planar sequence (TR/TE: 7000/85 ms); b-values: 50–1000 s/mm^2^
4	143	1.5-T scanner (GE Healthcare Life Sciences, Milwaukee, WI, USA)	Single-shot echo-planar sequence (TR/TE: 7000/85 ms); b-values: 0–900 s/mm^2^
5	107	3-T scanner (Achieva TX; Philips Healthcare, Best, The Netherlands)	Single-shot echo-planar sequence (TR/TE: 5336/ 61 ms); b-values: 0–800 s/mm^2^
6	88	3-T scanner (Trio Tim; Siemens Medical Systems, Erlangen, Germany)	Single-shot echo-planar sequence (TR/TE: 6600/91 ms); b-values: 0–1000 s/mm^2^

*Abbreviations: MRI* Magnetic resonance imaging, *DWI* Diffusion-weighted imaging, *TR* Repetition time, *TE* Echo time

### Statistical analysis

Continuous variables were described by mean value, median value, and SD. Categorical variables were given as relative frequencies. The comparison of ADC values in groups was performed by Mann-Whitney *U* tests where the *p* values are adjusted for multiple testing (Bonferroni correction). The association between ADC and Ki-67 values was calculated by Spearman’s rank correlation coefficient. Sensitivity, specificity, negative and positive predictive values, accuracy, and AUC were calculated for the diagnostic procedures. ADC thresholds were chosen to maximize the Youden index.

## Results

### ADC values and tumor grade/subtypes

**Fig. 1:**
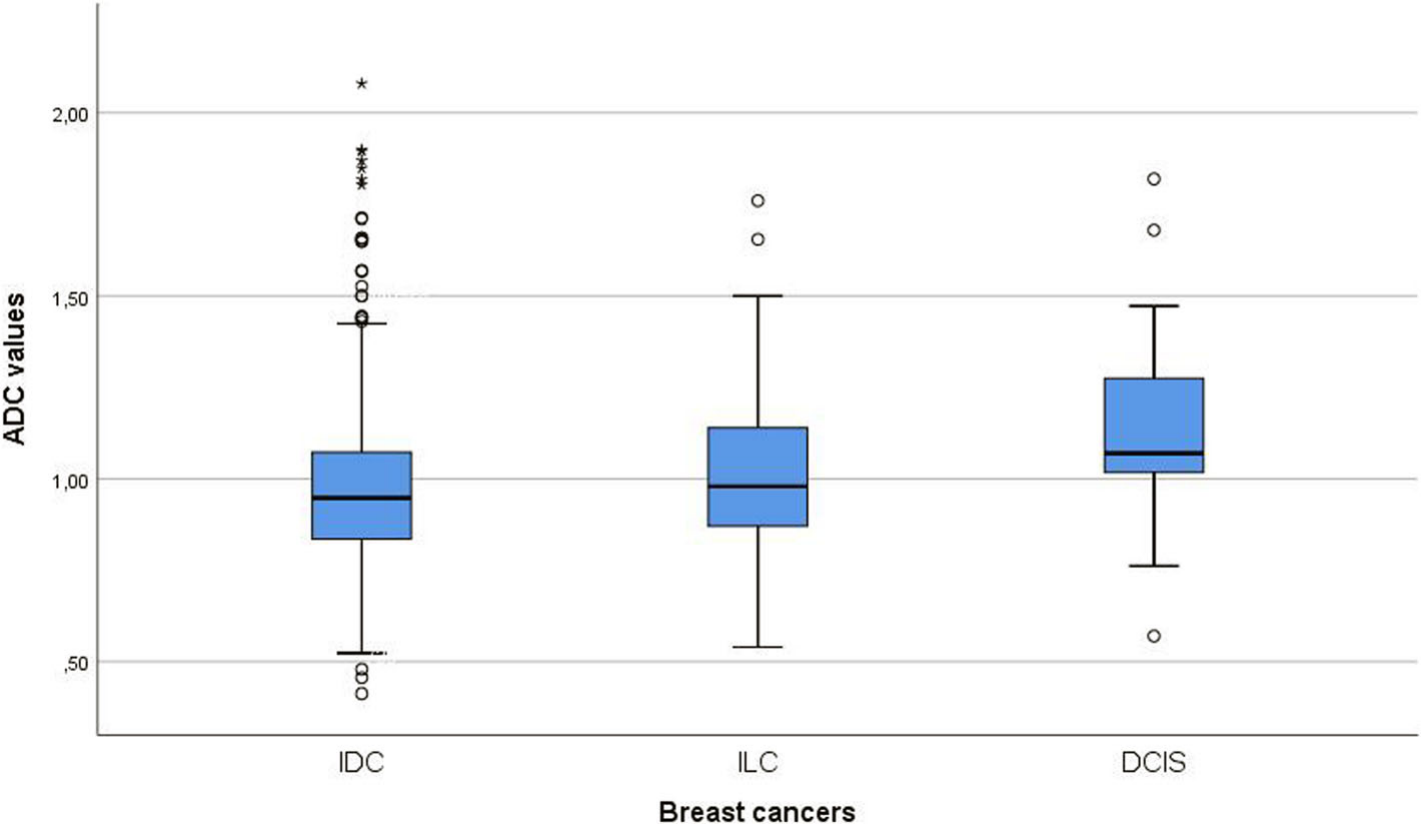
Comparison of apparent diffusion coefficient (ADC) values between ductal carcinoma in situ (DCIS), invasive ductal carcinoma (IDC) and invasive lobular carcinoma (ILC)

**Fig. 2:**
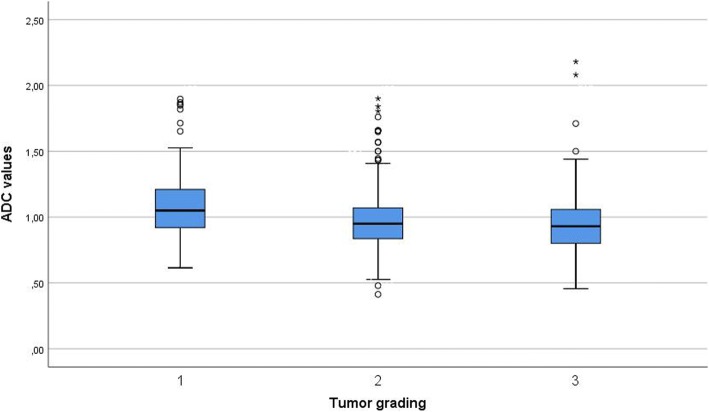
Comparison of apparent diffusion coefficient (ADC) values between grades 1, 2, and 3 tumors

Overall, data of 870 patients were acquired for this study. The majority of tumors were invasive ductal carcinoma (IDC; 81.95%), with a limited number of other subtypes (Table 1). The mean ADC value (× 10^− 3^ mm^2^ s^− 1^) of the tumors was 0.98 ± 0.22; the median value was 0.95; and the range was 0.41–2.18. Ductal carcinoma in situ (DCIS) showed statistically significant higher ADC values (1.11 ± 0.24 × 10^− 3^ mm^2^ s^− 1^) than IDC (0.97 ± 0.21 × 10^− 3^ mm^2^ s^− 1^; *P* = 0.001) and invasive lobular carcinoma (ILC; 1.01 ± 0.21 × 10^− 3^ mm^2^ s^− 1^; *P* = 0.044). There were no significant differences in ADC values between IDC and ILC (Fig. 1). Furthermore, ADC values differed between tumor grades. Grade 1 tumors had significantly higher ADC values (1.09 ± 0.27 × 10^− 3^ mm^2^ s^− 1^) than grade 2 (0.97 ± 0.21 × 10^− 3^ mm^2^ s^− 1^, *P* < 0.001) and grade 3 (0.95 ± 0.21 × 10^− 3^ mm^2^ s^− 1^, *P* < 0.001) lesions. No significant differences in ADC values were observed between grades 2 and 3 tumors (*P* = 1.00) (Fig. 2).

**Fig. 3:**
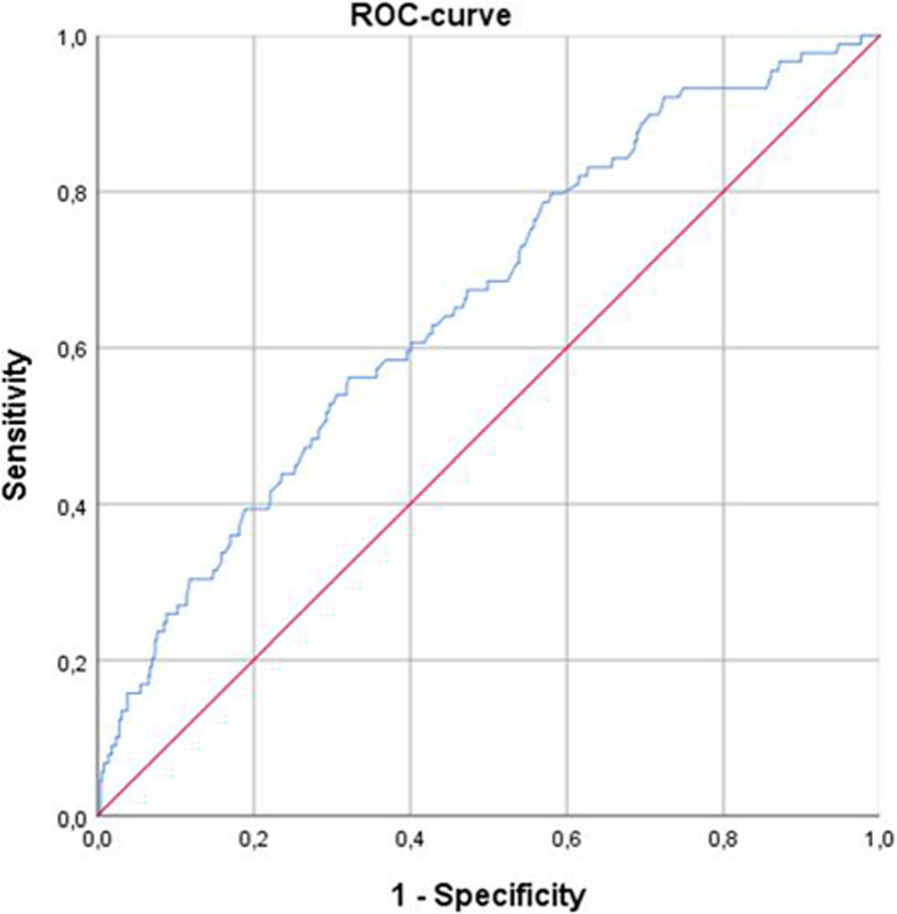
ROC curve for use of apparent diffusion coefficient (ADC) values in distinguishing grades 2 and 3 breast cancer from grade 1 tumors. Threshold ADC value = 1.03, sensitivity = 56.2%, specificity = 67.9%, positive predictive value = 18.2%, and negative predictive value = 92.4%

Next, ROC analysis was performed to differentiate high/moderate grade tumors from grade 1 lesions using ADC values. Youden index identified a threshold ADC value of 1.03 with a sensitivity of 56.2% and specificity of 67.9%. The positive predictive value was 18.2%, and the negative predictive value was 92.4%. ROC analysis (Fig. 3) showed that the AUC was 0.657.

### ADC values and Ki-67 level

**Fig. 4:**
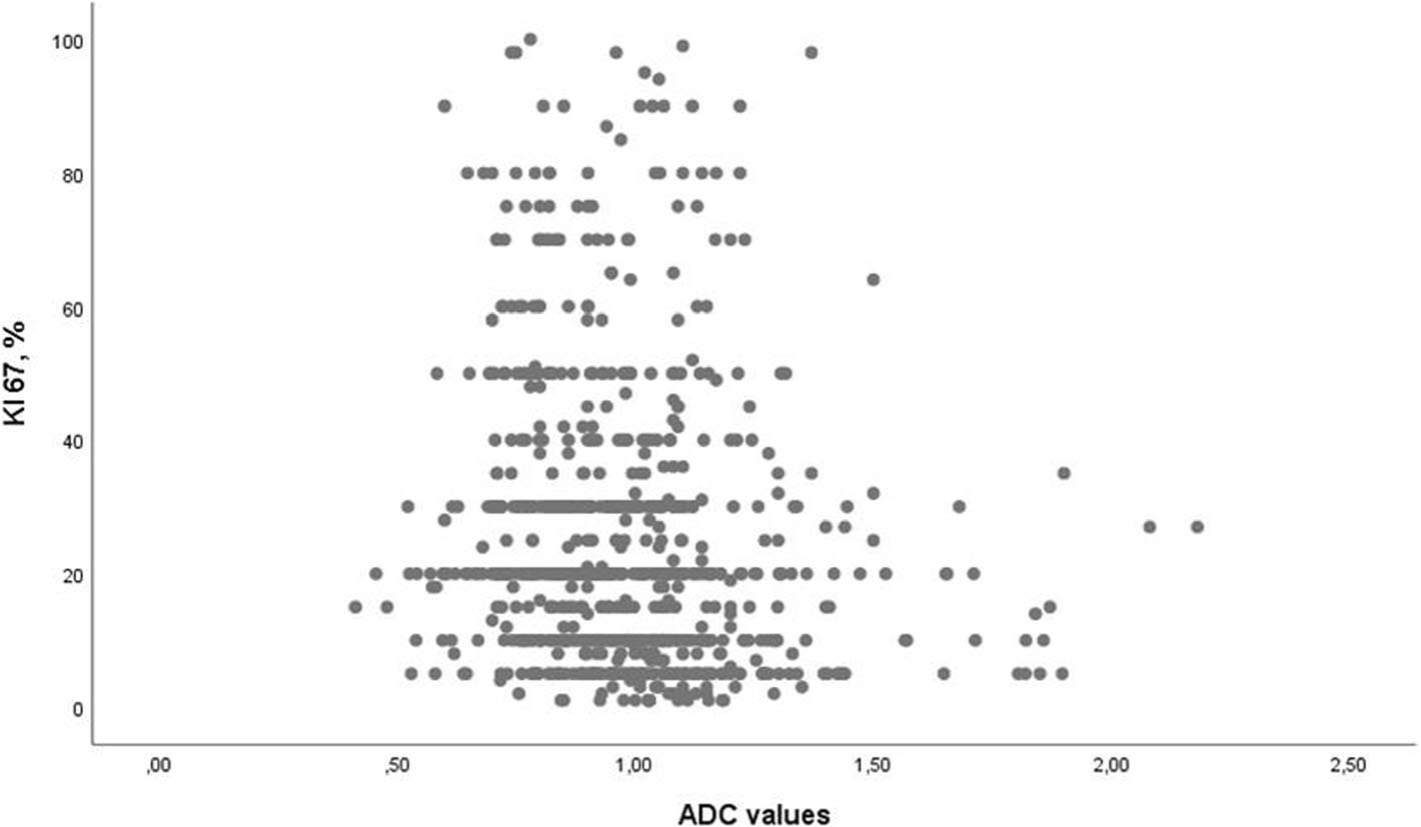
Correlation between apparent diffusion coefficient (ADC) and expression of Ki-67. The calculated correlation coefficient was − 0.202 (*P*< 0.001)

The level of the Ki-67 proliferation index was available for 845 patients. The mean value was 12.33 ± 21.77%; the median value was 30%; and the range was 1–100%. ADC correlated weakly with expression of Ki-67 (*p* = − 0.202, *P* < 0.001) (Fig. 4). Furthermore, ADC correlated with Ki-67 in the IDC subgroup (*p* = − 0.173, *P* < 0.001) and the ILC subgroup (*p* = − 0.296, *P* = 0.037), but not in the DCIS subgroup (*p* = 0.027, *P* = 0.859).

**Fig. 5:**
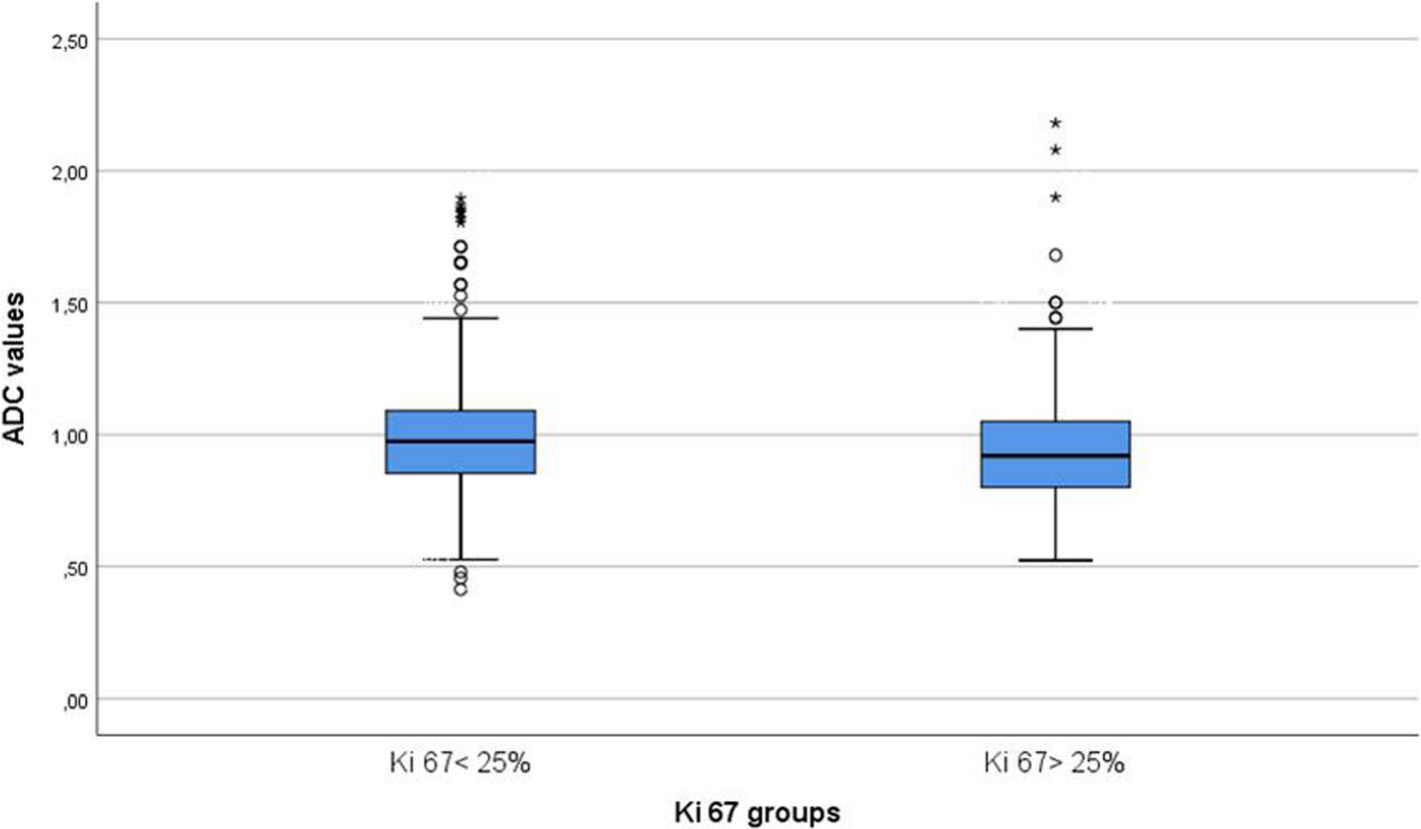
Comparison of apparent diffusion coefficient (ADC) values between tumors with low expression of Ki-67 (< 25%) and high expression of Ki-67 (≥ 25%)

A Ki-67 value of 25% was used as the threshold for discriminating between tumors with low Ki-67 expression (< 25%) and high Ki-67 expression (≥ 25%). Tumors with low expression of Ki-67 (*n* = 528 [62.49%]) had higher ADC values than tumors with high expression of Ki-67 (*n* = 317 [37.51%]) (0.99 ± 0.22 × 10^− 3^ mm^2^ s^− 1^ vs 0.95 ± 0.21 × 10^− 3^ mm^2^ s^− 1^, respectively; *P*= 0.005). However, ADC values of the subgroups overlapped significantly (Fig. 5).

**Fig. 6:**
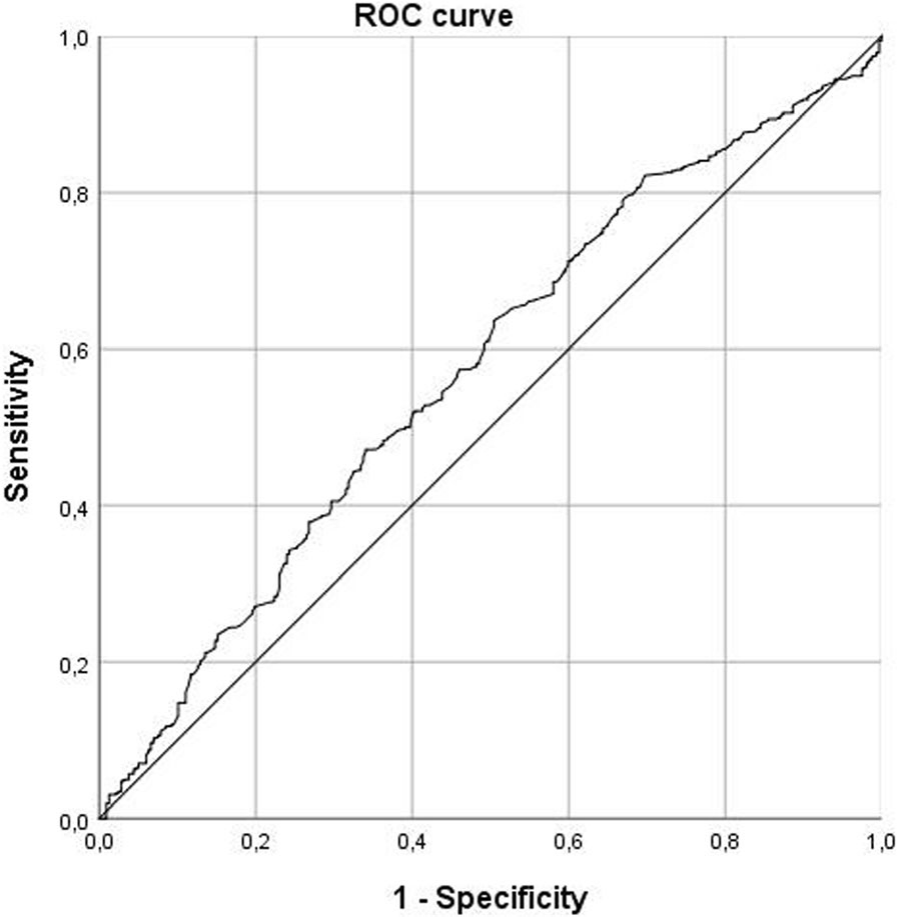
ROC curve for use of apparent diffusion coefficient (ADC) values in discrimination between tumors with low Ki-67 expression (< 25%) and high Ki-67 expression (≥ 25%). The threshold ADC value was 0.91. Sensitivity = 64.4%, specificity = 50.0%

In the next step, ROC analysis was performed to distinguish tumors with high proliferative potential from tumors with low expression of Ki-67 using ADC values. Youden index identified a threshold ADC value of 0.91. Using this threshold resulted in sensitivity of 64% and specificity of 50%. The positive predictive value was 67.7%, the negative predictive value was 45.0%, and the AUC was 0,574 (Fig. 6). Other threshold values of Ki-67 were also analyzed (*see* Table 3).

**Table 3:** Results of ROC analysis

Ki-67 expression threshold	ADC threshold	Sensitivity	Specificity	AUC	Positive predictive value	Negative predictive value
≥ 10%	0.951	0.704	0.542	0.644	0.267	0.885
≥ 20%	0.913	0.700	0.494	0.613	0.498	0.696
≥ 30%	0.913	0.639	0.512	0.590	0.712	0.430
≥ 40%	0.821	0.808	0.366	0.576	0.844	0.311
≥ 50%	0.821	0.807	0.413	0.600	0.892	0.263

*ADC* Apparent diffusion coefficient

## Discussion

This is the first multicenter study about relationships between ADC and histopathological features such as expression of Ki-67 and tumor grade in BC. Overall, it addresses a key question of whether imaging parameters can reflect clinically relevant histopathological findings. If so, then imaging, in particular ADC values, can be used as surrogate markers for tumor biology in BC.

Ki-67 is a well-established biomarker in BC [20, 21]. According to the literature, Ki-67 before and after neoadjuvant chemotherapy can predict the prognosis for patients with BC [21]. Furthermore, pretherapeutic Ki-67 is associated with pathological complete response after neoadjuvant chemotherapy in patients with BC [22]. In addition, Ki-67 is associated with overall and disease-free survival of patients with BC [23]. Therefore, it can be important in clinical practice to predict expression of Ki-67 on the basis of imaging.

ADC reflects diffusion of water molecules in tissue [24, 25]. Recently, a meta-analysis identified inverse correlations between ADC and cell count in several malignant and benign tumors [25]. Furthermore, it has been shown that ADC was associated with expression of Ki-67 in different lesions [26]. Several mechanisms may explain this association. Ki-67 is a nonhistone nuclear protein synthesized throughout the whole cell cycle except the G_0_ phase [27, 28]. Cytoplasmic proteins and cytoplasmic viscosity increase during mitosis [29]. These factors may influence water diffusion and ADC. Additionally, water diffusion may be affected by intracellular mitotic membranes.

As mentioned above, numerous prior studies investigated the role of ADC values in BC diagnosis and treatment. However, the reported results regarding associations between ADC and histopathology were inconclusive. Interpretation of prior results is complicated by differences in study design and analysis. The published radiological studies used different values of Ki-67 expression to discriminate tumors with low and high proliferative activity. For example, in the study of Zhuang et al., a Ki-67 level of ≥ 14% was considered to indicate high proliferation, and < 14% was considered to indicate low proliferation [30]. Some other studies used a threshold value of 20% [15, 16] or defined more than two Ki-67 categories. For example, De Felice et al. categorized Ki-67 expression as follows: low Ki-67 (≤ 14%), intermediate Ki-67 (15–30%), and high Ki-67 (≥ 30%) [13]. According to the meta-analysis of Petrelli et al., based on data of 64,196 patients, a Ki-67 cutoff > 25% is associated with a greater risk of death than lower expression rates [31]. Therefore, a reevaluation of the previous studies on associations between ADC and Ki-67 expression was needed.

The present study suggests that ADC cannot be used as a surrogate marker for proliferation activity in BC. In fact, although ADC values between tumors with high expression of Ki-67 (≥ 25%) differed from those with low levels of Ki-67 (< 25%), the calculated specificity and sensitivity were too low. This applied also for several alternative thresholds of Ki-67 expression ranging from 10% to 50%. Similar results were also observed for distinguishing low-grade and intermediate/high-grade tumors. Statistical analysis identified that grade 1 lesions had higher ADC values than grades 2 and 3 tumors. However, the ROC analysis showed that a possible use of ADC for discrimination of tumor grade in BC has very low specificity and sensitivity. Furthermore, we found that DCIS had statistically significant higher ADC values than IDC and ILC. However, ADC values also overlapped also overlapped distinctly in these subtypes. Therefore, use of ADC does not provide specific information regarding tumor biology in BC that can be used reliably in clinical practice.

The present study is the largest to date on this topic, to our knowledge. However, it has some limitations. The involved patients were investigated with use of different MRI equipment with different technical parameters, such as field strength, DWI sequences, and b-values. This may broaden the range of ADC values in the study and may have influenced our results. Furthermore, the patient samples consisted predominantly of only three tumor subgroups, namely DCIS, IDC, and ILC. Our study identified that associations between ADC and Ki-67 were different in several subtypes of BC. Moreover, the calculated correlation coefficients for IDC, DCIS, and ILC in our study differed significantly from those for mucinous carcinoma (*r* = 0.035, *p* = 0.892) reported by Onishi et al. [32]. Presumably, associations between ADC and Ki-67 or tumor grade may be different in other subtypes of BC such as tubular or medullary carcinomas. However, the included tumors represent the most frequent subtypes of BC, whereas other carcinomas are rarer. We did not analyze possible associations between ADC and other clinically relevant biological features in BC, such as hormonal receptor status and/or HER2 status. This interesting aspect is a goal of further studies.

## Conclusions

Our multicenter study shows that ADC cannot be used as a reliable surrogate marker for proliferative activity and/or for tumor grade in BC.
